# Effectiveness of electronic patient reporting outcomes, by a digital telemonitoring platform, for prostate cancer care: the Protecty study

**DOI:** 10.3389/fdgth.2023.1104700

**Published:** 2023-05-08

**Authors:** C. Helissey, C. Parnot, C. Rivière, C. Duverger, A. Schernberg, S. Becherirat, H. Picchi, A. Le Roy, P. Vuagnat, R. Pristavu, H. Vanquaethem, L. Brureau

**Affiliations:** ^1^ Clinical Research Unit, Department of Oncology, Military Hospital Begin, Saint-Mandé, France; ^2^ Department of Medical Oncology, Military Hospital Begin, Saint-Mandé, France; ^3^ Clinical Research Department, Cureety, 33 rue de l’Amirauté, Dinan, France; ^4^ Department of Internal Medicine, Military Hospital Begin, Saint-Mandé, France; ^5^ CHU de Pointe-à-Pitre, Univ Antilles, Univ Rennes, Inserm, EHESP, Irset (Institut de Recherche en Santé, Environnement et Travail)—UMR-S 1085, Pointe-à-Pitre, France

**Keywords:** e-PRO, prognostic factor, prostate cancer, health care, quality of life

## Abstract

**Research aim and purpose:**

The benefits of Electronic Patient -Reported Outcomes (e-PRO) for telemonitoring are well established, allowing early detection of illnesses and continuous monitoring of patients. The primary objective of the PROTECTY study was to assess the compliance with patient use of the telemonitoring platform Cureety. An exploratory objective was to assess if the first-month health status is a prognostic factor of progression free-survival (PFS) and overall survival (OS) for prostate cancer patient.

**Methods:**

This prospective study was conducted at the Military Hospital Bégin on prostate cancer patients. Patients were allowed to respond to a symptomatology questionnaire based on CTCAE v.5.0, personalized to their pathology and treatment. An algorithm evaluates the health status of the patient based on the reported adverse events, with a classification into 2 different states: Good Health Status (GHS) and Poor Health status (PHS).

**Results:**

Sixty-one patients were enrolled between July 1st, 2020 and September 30th, 2021. The median age was 74.0 (range 58.0–94.0). 78% presented a metastatic stage, and the most represented cancer was mHSPC. Overall, 2,457 questionnaires were completed by the patients, 4.0% resulted in a health classification in to monitor or critical state. 87% of patients were classified in the GHS group. The compliance was 72% in the overall population during the first month, 71% in GHS group and 75% in PHS group. The median follow-up was 8 months. PFS at 6 months was 84% in GHS group vs. 57% in PHS group, *p* = 0.19. OS at 6 months was 98% in GHS group vs. 83% in PHS group, *p* = 0.31.

**Conclusions:**

Our study showed that compliance was satisfactory. The feasibility of remote monitoring for prostate cancer patients means that they should benefit from its implementation. Our study is also the first to assess the correlation between treatment tolerance and survival. The initial results suggest that e-PRO assessment could help identify in the early stages the patients that require further health assessment and potential therapeutic changes. While further follow-up of more patients will be required, our study highlights the importance of e-PRO in cancer patient care.

## 1. Introduction

Prostate cancer is the second most common cancer affecting men worldwide, with 1.41 million cases in 2020 according to the World Health Organization, and is responsible for 375,000 deaths every year (1).

In recent years, the management of prostate cancer has changed dramatically, leading to a marked improvement in patient survival. New treatments have emerged, with new hormonotherapy options, PARP inhibitors, and metabolic therapy (2–7). The development of these treatments initially in monotherapy and later in combination therapy has clearly improved the survival of patients at first in the metastatic stage and today in the localized stage (8).

However, these innovative treatments are responsible for adverse events which can impact the quality of life of patients. Clinicians often underestimate the side effects of these treatments in relation to the patients' feelings and therefore have a false perception of their quality of life (9). As a result, many adverse events are under-recognized, under-reported and therefore under-treated (10).

Electronic Patient Remote Outcomes (e-PRO) allow to obtain the patient's perception directly, without any interpretation of their answer, using a validated questionnaire (11). Thus, e-PRO reflect the impact of not only the disease but also the treatments, on the quality of life of the patients.

The benefits of e-PRO-based remote monitoring are widely recognized, for enabling physicians to understand the patient experience, detecting disease early and in real-time, preventing disease progression and premature death, and reducing hospital costs and hospitalizations (12). In addition, e-PRO remote monitoring provides more accurate records of patients' daily activities, thus improving the efficiency of healthcare delivery through the use of digital communications and emergency medical care when needed (12).

Remote monitoring has known benefits for patients with chronic illnesses including diabetes, psychiatric and cardiovascular diseases, and cancer (13–15). These benefits extend beyond clinical outcomes to medico-economic gains (16). For instance, a meta-analysis by Kim et al. found that remote monitoring was associated with a significant decrease in glycated hemoglobin levels in patients with type 2 diabetes compared to standard care (17). The widespread use of connected objects has facilitated the implementation of telemedicine in practice (18, 19). This technology enables direct monitoring of patient tolerance to treatment, bridging the gap between patient perception and care team interpretation of adverse events. Moreover, telemonitoring has therapeutic and psychological benefits for patients and enhances treatment adherence. Symptom monitoring was shown by Basch et al. to improve the quality of life of cancer patients (20). Remote monitoring can also improve overall survival in patients with bronchial cancer, as demonstrated by Denis et al., who reported a 68% reduction in mortality risk among patients who used a remote monitoring platform (21). Furthermore, telemedicine can reduce geographic inequalities in access to care, as noted by Russo et al., who reported travel time savings and cost savings due to the use of telemedicine (16).

However, its routine use remains limited. Some of the barriers to adoption are technical or connectivity issues experienced by patients, uncertainty in the language used, patient adherence to these telemonitoring platforms, especially for older patients, and the correct use of the data in current practice, in particular choosing a questionnaire corresponding to the actual patient's needs (22, 23). In a previous study, we evaluated the usefulness of the digital platform Cureety for the remote monitoring of older cancer patients, and showed that compliance with the use of the digital tool was high in that subset of patients (24). However, to our knowledge, there is no study focused on the impact of remote patient monitoring on prostate cancer.

Remote monitoring could help address several of the challenges specific to the current management of prostate cancer patients, such as the monitoring of new hormonal therapies, ensuring compliance, and having a better view of adverse events experienced by the patients. Such a close monitoring would also allow an early therapeutic adaptation depending on the clinical state of the patient, even with oral therapies where there is typically less monitoring.

To explore these questions, the PROTECTY study is focused specifically on prostate cancer patients. Building from the lessons learned from our previous study, this new study aims to evaluate the compliance with the tool of prostate cancer patients, and to evaluate the prognostic power on survival of their health status during the first month of treatment.

## 2. Patients and methods

The EPROTECTY study is an observational prospective study, conducted in the Clinical Research Unit in Military Hospital Bégin. The study was conducted in accordance with Good Clinical Practice and the Declaration of Helsinki. The study was approved by a local Ethics Committee.

### 2.1. Patients

The study was conducted from July 1, 2020 to May 30, 2022. All prostate cancer patients treated at the Bégin Military Hospital were eligible to participate in the study. There were two exclusion criteria: minors (17 years or younger) and patients who did not consent to the use of a digital remote monitoring tool. Patients were included during hospital visits while receiving anticancer therapy. Patients with internet access *via* smartphone or computer were included in the “app monitoring” cohort. Patients with no internet access or low digital autonomy were included in a “phone monitoring” cohort and were regularly contacted by phone to answer a personalized questionnaire assigned to them. All patients included in the PROTECTY study signed informed consent. We worked in part from patients from a previous study (24), but we focused here on prostate cancer patients, included new patients and were able to analyse the data over a longer monitoring period for all patients, compared to our previous work.

### 2.2. Study design

Each cancer patient could complete a personalized symptom questionnaire tailored to their pathology and treatment using a digital remote monitoring platform called Cureety, as described previously (18).

The questionnaire ranked adverse events (AEs) related to the patient pathology and treatment following the CTCAE (Common Terminology Criteria for Adverse Events). For each completed questionnaire, the algorithm calculates a global health score to place the patient in one of four different states: Correct (green), Compromised (yellow), To be monitored (orange), or Critical (red). The classification or its color is not shown directly to the patient, but in the case of green or yellow status, the patient only receives treatment advice on how to manage their AE. For orange or red status, patients also receive treatment advice but are also told to call the hospital or their GP.

For the purposes of this study, we calculated two endpoints, “Compliance” and “First-Month Tolerance”. The compliance with the digital remote monitoring tool indicates whether patients are responding to digital questionnaires as often as expected (once a week for chemotherapy, once every two weeks for hormonotherapy or targeted therapies). Tolerance in the first month indicates whether the patient tolerates the first 30 days of treatment. To do this, we calculated the number of days the patient's health score was green or yellow (count A) and the number of days the patient was orange or red (count B), during the first 30 days of monitoring. If A was higher than B, the patient was classified as “healthy” (GHS group), otherwise it was classified as “poor health” (PHS group).

**Figure 1:**
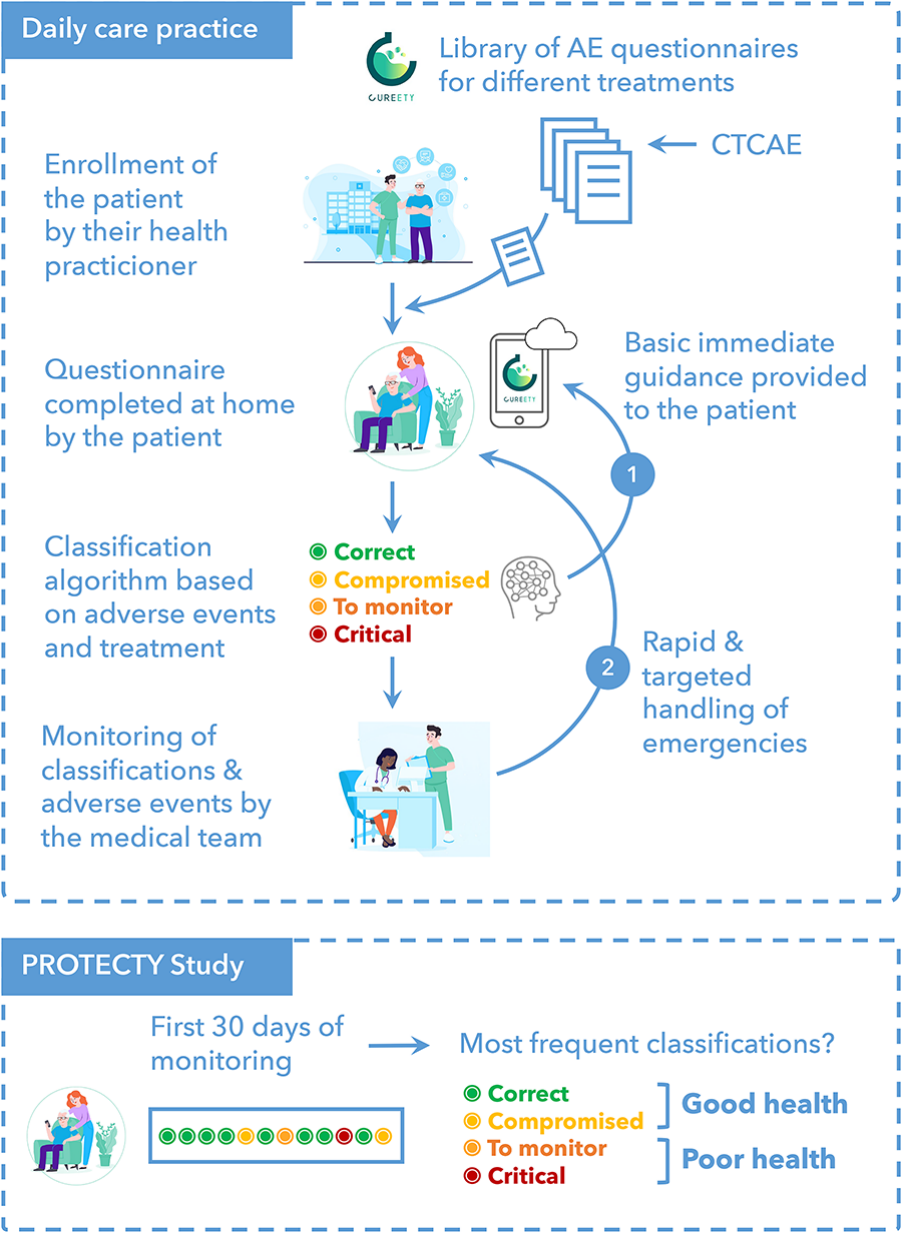
Study design.

The study design is presented in Figure 1.

The primary endpoint was to assess the compliance with a telemonitoring platform among prostate cancer patients in order to optimize patient care.

The exploratory endpoint was to assess if the first-month health status was a prognostic factor of progression free-survival (PFS) and overall survival (OS).

### 2.3. Data collection and measurements

Population statistics (age at inclusion, gender, comorbidities, performans status, weight, height), disease characteristics (primitive, histology, stage at platform's inclusion, molecular biology), therapeutic characteristics (type, duration), enrollment in clinical trials were collected. Individual AEs, grades reported by patients, and global health status were recorded using the digital remote monitoring tool Cureety.

The digital platform Cureety allows remote patient monitoring. It is a CE-marked medical device that was designed and tested for its use by cancer patients, including older patients (24). The platform has been used as part of routine care at the Bégin hospital since July 2020, as described previously (14–24). It has been used by several hospitals in France for the monitoring of more than 2000 cancer patients over the past 3 years. During the study, each cancer patient was allowed to respond to an AE questionnaire personalized to their pathology and treatment. The questionnaires follow the CTCAE (Common Terminology Criteria for Adverse Events) to grade AEs. Patients could answer using a mobile device or a computer, on their own schedule, up to once a day. For each completed questionnaire, a global health score was computed to classify the patients into one of four different states: Correct (green), Compromised (yellow), Fragile (orange) or Critical (red). Patients were not explicitly shown the result, but in the case of green or yellow classifications, they received only. In the case of orange or red classifications, they receive therapeutic recommendations and are invited to call their medical team. In all cases, they received therapeutic recommendations to manage their AEs.

### 2.4. Statistical analysis

Baseline demographics and digital background were summarised with descriptive statistics. Survival probabilities were estimated by the Kaplan–Meier method. Progression-free survival (PFS) was deﬁned as the time from inclusion to progression; and overall survival (OS) was deﬁned as the time from inclusion to all-cause death. All statistical analyses were carried out with Statview software (SAS Institute, Cary, NC). All tests were two-tailed, and *p* values lower than 0.05 were considered significant (Supplementary Material).

## 3. Results

### 3.1. Patient characteristics

Sixty-one patients were enrolled between July 1st, 2020 and September 30st, 2021. The median age was 74 (range 58–94), with more than 67% of the patients over the age of 70. Fourty-seven patients (78%) presented a metastatic stage, and the most represented cancer was mHSPC (77%).

Forty-eight patient (79%) presented at least one comorbidity. Fourty-six patients (75%) received new hormonal therapy, 13 patients (21%) received chemotherapy and 2 patients (3%) received a combination treatment.

We calculated the tolerance of patients during their first month of treatment, based on the health classification computed by the telemonitoring tool (see methods). Fifty-three patients (87%) were classified GHS and 8 patients (13%) were classified PHS.

Baseline characteristics are summarized in Table 1.

**Table 1:** Baseline patients’ characteristics.

Variables	Total
Number of patients (%)	61 (100%)
	**Median (range)**
Age at inclusion (years)	74.0 (58.0–94.0)
Follow-up (months)	8.1 (0.5–14.2)
	**Number (%)**
Performans status 0–1	61 (100%)
**Age (years)**	
<70	20 (32.8)
≥70	41 (67.2)
**Comorbidities**	
No	13 (21.3)
Yes (at least one)	48 (78.7)
**Types of treatment**	
Chemotherapy	13 (21.3)
Hormonotherapy	46 (75.4)
Combined treatment	2 (3.3)
**Stage at inclusion**	
Localized disease	13 (21.7)
Advanced disease	47 (78.3)
**Metastatic**	
mHSPC	36 (76.6)
mCRPC	11 (23.4)

### 3.2. Patient-reported outcomes on AEs

**Figure 2:**
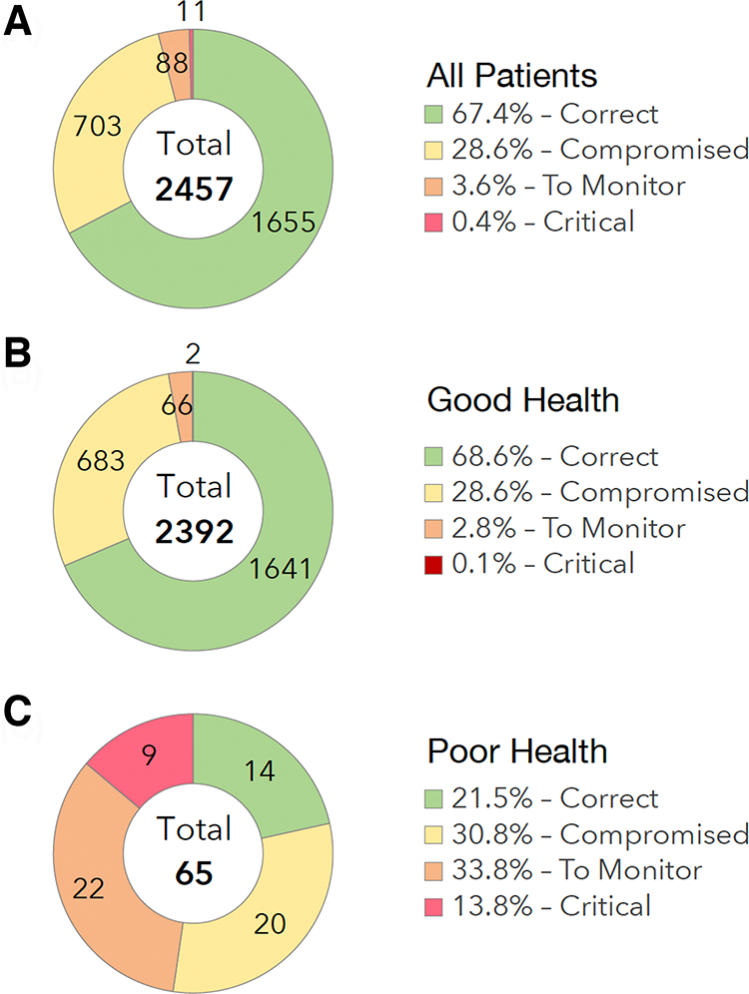
Patient reported outcomes. (**A**) All Patients. (**B**) Good Health Status. (**C**) Poor Health Status.

Out of 2,436 ePRO questionnaires completed by the patients, 67% (*n* = 1,655) corresponded to a “correct” state, 29% (*n* = 703) to a “compromised” state, 4% (*n* = 88) to a state “to be monitored” (Figure 2A). The main adverse events reported by the patients were asthenia (50.8%), joint/muscle pain (50.8%) and hot flushes (37.7%).

Out of 2,392 ePRO questionnaires completed by the patients in GHS group, 69% (*n* = 1,641) corresponded to a “correct” state, 29% (*n* = 683) to a “compromised” state, 3% (*n* = 66) to a state “to be monitored” (Figure 2B).

Out of 65 ePRO questionnaires completed by the patients in the PHS group, 21% (*n* = 14) corresponded to a “correct” state, 31% (*n* = 20) to a “compromised” state, 34% (*n* = 22) to a state “to be monitored”, and 14% (*n* = 9) to a “critical” state (Figure 2C).

### 3.3. Compliance

The average patient adherence to weekly or bi-weekly completions was 72% during the first month, and over the whole duration of the patient monitoring (median follow-up of 8 months), the adherence was 59% (Table 2).

**Table 2:** Compliance to the digital plateform cureety over time.

	All patients	GHS	PHS
Number of patients (*N*, %)	61 (100%)	53 (86.9%)	8 (13.1%)
Compliant patients—1 month (*N*, %)	44 (72.1%)	38 (71.7%)	6 (75%)
Compliant patients—6 months (*N*, %)	36 (59%)	33 (62%)	3 (37.7%)

### 3.4. Tolerance

**Figure 3:**
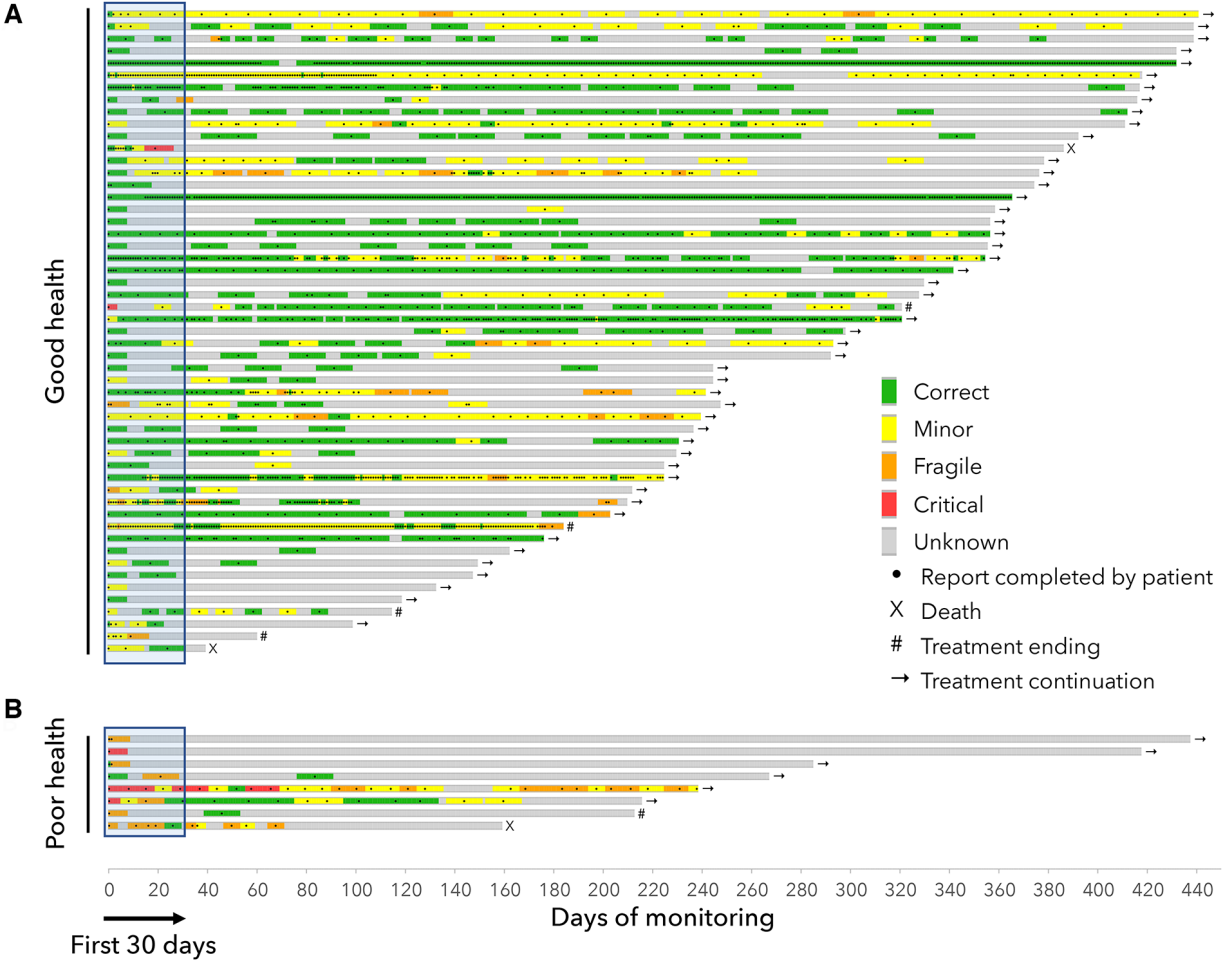
Timelines for patients in the “Good health” group (**A**) and in the “Poor health” group (**B**) Each line represents the monitoring of a patient and shows the clinical classifications computed by the device algorithm (green/yellow/orange/red) from the completed questionnaires (black dots). The end of each timeline correspond to either the end of the study, the end of the treatment or death.

**Figure 4:**
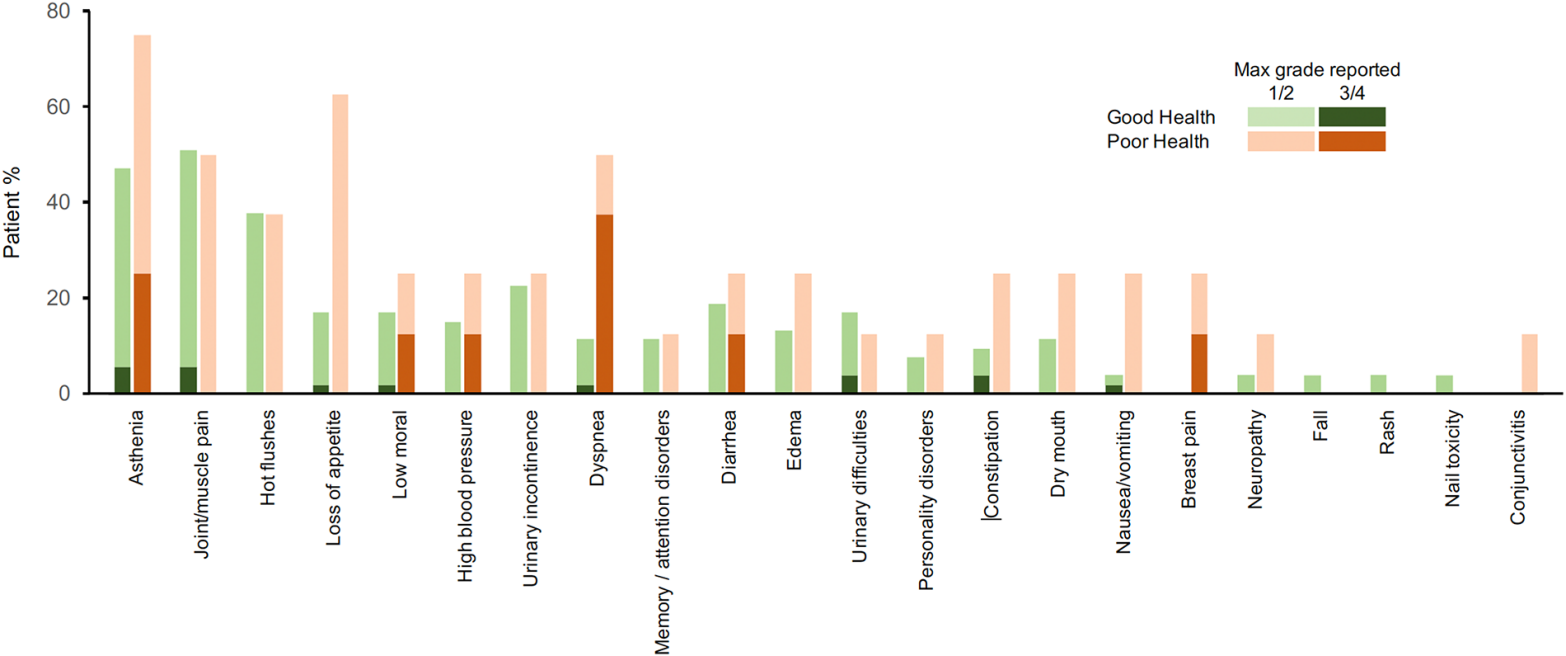
Safety profile. Reports of adverse events with grade 1–4 in the first 30 days of monitoring.

The Figure 3 displays the tolerance's timelines for each patient. In the GHS group, the main adverse events reported by the patients were joint/muscle pain (51% of patients for all grades, and 5.7% for grades 3 or 4), asthenia (47% all grades, 5.7% grades 3 or 4), hot flushes (38% all grades, 0% grades 3 or 4). In the PHS group, the main adverse events reported by the patients were asthenia (75% all grades, 25% grades 3–4), loss of appetite (62% all grades, 0% grades 3–4), dyspnea (50% all grades, 37.5% grades 3–4) and joint/muscle pain (50% all grades, 0% grades 3–4) (Figure 4).

### 3.5. Progression free survival (PFS) and overall survival (Os) correlated with the health status

**Figure 5:**
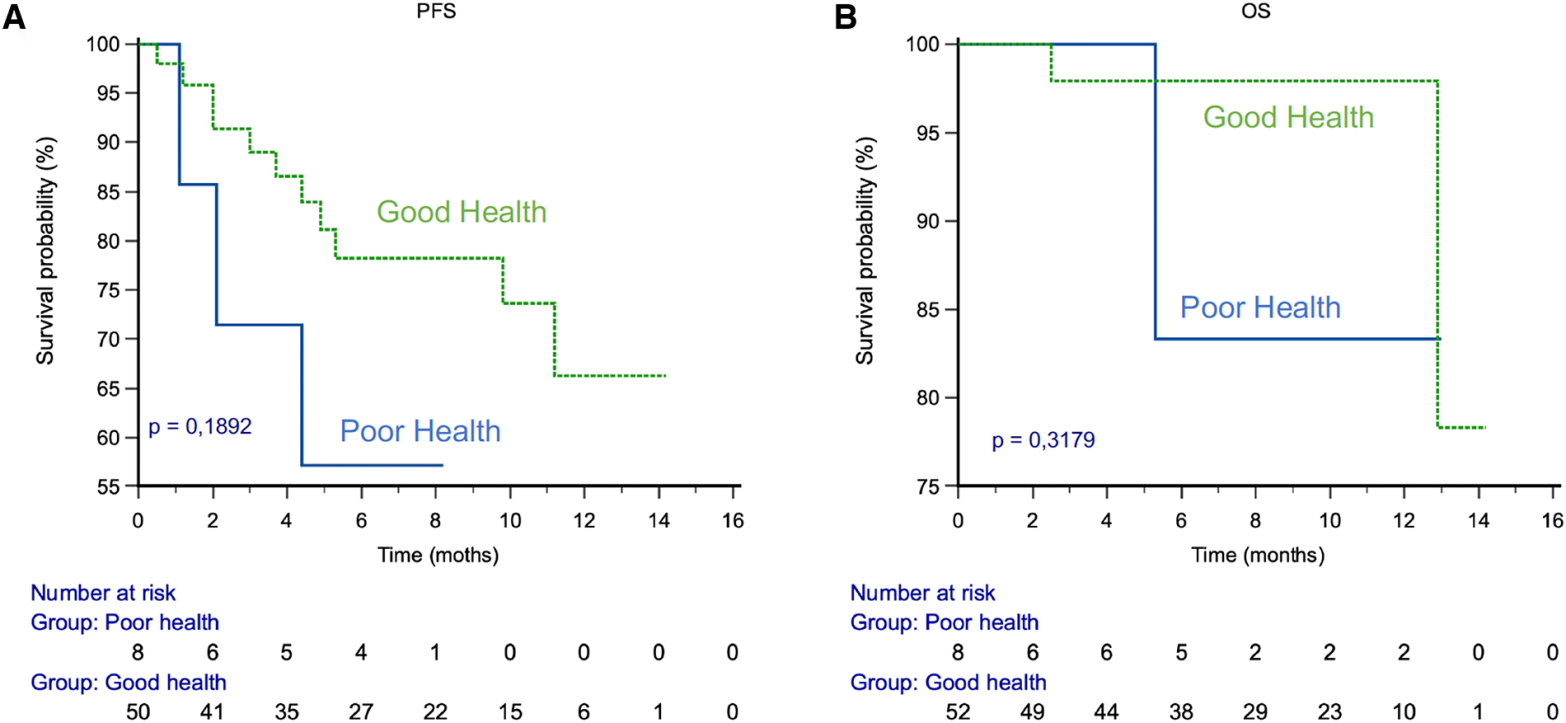
PFS and OS by health status. (**A**) PFS. (**B**) OS.

After a median follow up of 8 months, PFS at 6 months was 89% in the GHS group vs. 60% in the PHS group, *p* = 0.17. OS at 6 months was 100% in GHS group vs. 86% in the PHS group, *p* = 0.15 (Figure 5).

## 4. Discussion

The PROTECTY study provides valuable insights into the potential of e-PRO assessment for cancer patient care. In this study, we hoped to address some of the challenges specific to this pathology. In particular, the monitoring of oral therapies or combinations at the onset of prostate cancer remains particularly challenging. The ability to better monitor compliance and adverse events in real time should significantly improve the quality of care for the patients. To explore these questions, we used a subset of a cohort of cancer patients from a previously published study, with the inclusion of new patients and a longer monitoring period (24).

The compliance with the digital platform was 72% during the first month, suggesting that e-PRO assessment is acceptable for most patients.

Our study is one of the first to investigate the relationship between the e-PRO measurement of postate cancer patients’ health status and their clinical outcomes.

The study found a correlation between the health status of patients during the first month of treatment and their survival. The study identified 4.0% of patients in a monitor or critical state, indicating the need for further health assessment and potential therapeutic changes.

The safety profile reported in most studies derives from the interpretation of the clinician based on the CTCAE scale. However, there is a clear difference between the patient's experience of toxicity and its grading by the practitioner. It is therefore essential to obtain data directly from the patient. Based on this observation, we chose to rely on patient reported outcomes (PRO), which are defined as any report of a patient's health status that comes directly from a patient without interpretation by a clinician or any other person (11).

These, based on the validated scales defined by the CTCAE (25, 26), allow to evaluate the quality of life of the patient, through their physical, psychological, social and functional state, and thus to evaluate the impact of not just the disease but also the treatnent, on their quality of life.

Both the FDA and the EMA have recognized that obtaining good quality data from patient-reported outcome measures (PROMs) is important when evaluating drugs in patients for whom palliation of symptoms is an important therapeutic goal; consequently, both agencies have published guidance and recommendations for the use of PROMs in clinical trials (10, 11, 27, 28). With over 90% of patients owning mobile phones and 87% using the Internet, the increasing use of connected objects facilitates the wide adoption of telemedicine and of e-PRO collection (18, 19).

Beyond the reporting of side effects experienced by the patient, the benefits of remote monitoring are well documented, especially with a clear improvement in the quality of life of cancer patients. Basch et al. measured the impact of symptom monitoring on the management of 766 cancer patients and found significant improvements in their quality of life thanks to remote monitoring (34% vs. 18%, *p* < 0.001) (20).

Remote monitoring has also been shown to improve overall survival in cancer patients. Denis et al. evaluated the impact of the remote monitoring platform on overall survival of patients with bronchial cancer compared to standard practice (21). The mortality risk was reduced by 68% in the patients monitored with the platform [hazard ratio = 0.32, 95% CI (0.15 to 0.67), *p* = 0.002] (30). Remote monitoring also has medico-economic benefits. Russo et al. reported an average savings of $18,555 per year in travel costs (16).

They allow for a better patient-provider relationship, and early detection of side effects improves their management.

The benefits of remote monitoring for cancer patients are well documented, both in terms of quality of life and survival. Remote monitoring improves the patient care pathway while maintaining contact with ambulatory patients and anticipating the management of adverse events at an early stage to avoid serious deterioration.

Despite these benefits, the use of e-PRO remains limited in current practice. Various prejudices may hinder its use, such as an underestimation of patient adherence to this mode of monitoring, the limited interest in our current practice (22).

However, few studies have evaluated the feasibility of remote monitoring in patients with prostate cancer, especially in the metastatic stage, where the majority of treatments received by these patients cause fatigue and impact their quality of life.

The main objective of the PROTECTY study was to evaluate compliance of prostate cancer patients with a digital remote monitoring platform.

Here we demonstrated strong adherence to a digital remote monitoring platform, with 72% of compliance during the first month. This is an essential first step before we can expect any benefit from remote monitoring. Tran et al. confirmed the high acceptability (29). They reported that the use of a telemonitoring app, using ePROs, was feasible and acceptable in patients with localized or advanced prostate cancer. Patients reported that use of the smartphone app was easier or equivalent to the traditional paper-and-pencil approach, demonstrating acceptability and support for the use of remote monitoring of PROs (29).

As part of the larger cohort from which the subset of prostate cancer patients was extracted for this study, we had previously shown a similar strong adhesion in older patients, as 70% of them were compliant and 72% of the patients were satisfied (24). However, we also observed a drop in patient compliance over time. There is likely room for improvement in the functioning of our technology in order to maintain patient adherence over time and maintain the benefits of remote monitoring.

Moreover, we have demonstrated in this larger cohort that the use of e-PRO allows to measure a health-related quality of life, not just a collection of adverse events, thus better estimating the quality of life of the patient with his disease and his treatment. Furthermore, 67% of the patients were classified in a “correct” state.

But beyond that, it can be a tool for therapeutic management. Kerrigan et al. reported that the baseline clinical assessment of patients by PROs may be prognostic of both cancer survival and likelihood of hospitalization (30). Movsas et al. confirmed the importance of baseline quality of life assessment, using the QLQ30 scale, in patients with locally advanced non-small cell lung cancer (31). And this factor is a more performant factor than the classical performans status. This is helpful for the initial therapeutic strategy, and therefore the choice of therapy.

It is also important to follow the tolerance of the treatment over time via the PRO-CTCAE. Indeed, our study shows that the patient's health status would be a prognostic factor for survival. Progression-free survival is 29% higher at 6 months in the GHS group compared to the PHS group. Similarly, overall survival is 14% higher at 6 months in the GHS group compared to the PHS group.

Cella et al. recently reported on the relationship between health-related quality of life (HRQoL) and clinical outcomes in patients with advanced renal cell carcinoma (aRCC) in the CheckMate (CM) 214 study (32). It reported a stronger association for longitudinal health-related quality of life (HRQoL) with OS compared with the baseline HRQoL model. Thus, HRQoL responder patients had better overall survival compared to HRQoL non-responders, with a 52% reduction in mortality risk in HRQoL responders [HR = 0.48 (0.39–0.59), *p* < 0.0001] (32).

These two studies underline the importance of telemonitoring in the management of patients and to anticipate iconographic assessments in PHS patients, in order to avoid subjecting them to ineffective and poorly-tolerated treatments.

As part of an exploratory analysis, we also determined whether the health status of our patients at month 1 (GH = “Gooh Health”, or “Poor Health” = PH, as evaluated by the monitoring algorithm) predicted their survival. The numerical figures showed that GH patients have a better survival than PH, but the difference between the two groups was not significant. The lack of significance was related to the small sample size, in particular in the PH group. Based on these initial promising results, we plan to conduct a larger study to evaluate this impact as a primary objective.

Despite the promising results of the PROTECTY study, there were several limitations to the study design that should be taken into consideration. Firstly, the study was conducted on a relatively small sample size of 61 patients, which may limit the generalizability of the findings. Secondly, the study was conducted at a single center, which may limit the applicability of the results to other settings. Finally, the follow-up period of 8 months was relatively short, and longer follow-up periods are necessary to determine the long-term impact of e-PRO assessment on patient outcomes.

Despite the limitations of the PROTECTY study, the results suggest that e-PRO assessment could be a valuable tool for identifying patients who require further health assessment and potential therapeutic changes. Future studies should aim to replicate the findings of the PROTECTY study on larger sample sizes and in multiple centers to determine the generalizability of the findings.

Additionally, studies should be conducted on patients with different types of cancer to determine the applicability of e-PRO assessment to different patient populations. Finally, longer follow-up periods are necessary to determine the long-term impact of e-PRO assessment on patient outcomes, such as overall survival and quality of life.

Although our study did not show significant differences between the two groups, given the size of the sample, it highlights the importance of telemonitoring. Patients are satisfied with the system and adhere to the digital platform.

## 5. Conclusion

Our study is the first to assess the impact of tolerance treatment on survival, using the first-month health status from the telemonitoring platform for prostate cancer patients.

The initial results suggest that e-PRO assessment by the platform could help identify in the early stages the patients that require further health assessment and potential therapeutic changes.

The PROTECTY study provides a valuable insight into the potential benefits of e-PRO assessment for cancer patient care, but further research is needed to fully understand its potential impact on patient outcomes.

## Data Availability

The raw data supporting the conclusions of this article will be made available by the authors, without undue reservation.
