# Supplementary material for: Effectiveness of electronic patient reporting outcomes, by a digital telemonitoring platform, for prostate cancer care: the Protecty study
Source: Front Digit Health. 2023 May 8;5:1104700. doi: 10.3389/fdgth.2023.1104700 (PMC10203955; doi:10.3389/fdgth.2023.1104700)
Supplement: Supplementary file 1 [file Image1.pdf]

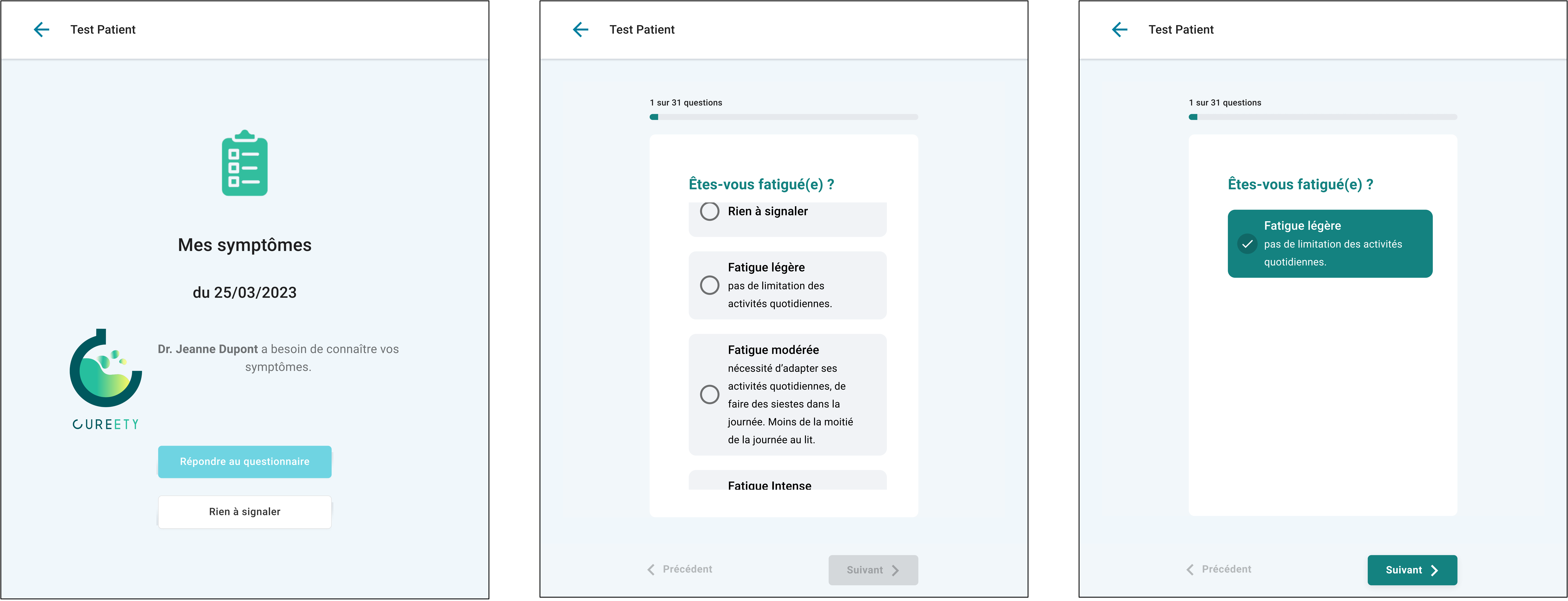

Supplementary Figure 1.
Patient interface of the Cureety application, showing an example of a question included in the AE questionnaire used to evaluate the patient's health.
