# Supplementary material for: Effectiveness of electronic patient reporting outcomes, by a digital telemonitoring platform, for prostate cancer care: the Protecty study
Source: Front Digit Health. 2023 May 8;5:1104700. doi: 10.3389/fdgth.2023.1104700 (PMC10203955; doi:10.3389/fdgth.2023.1104700)
Supplement: Supplementary file 2 [file Image2.pdf]

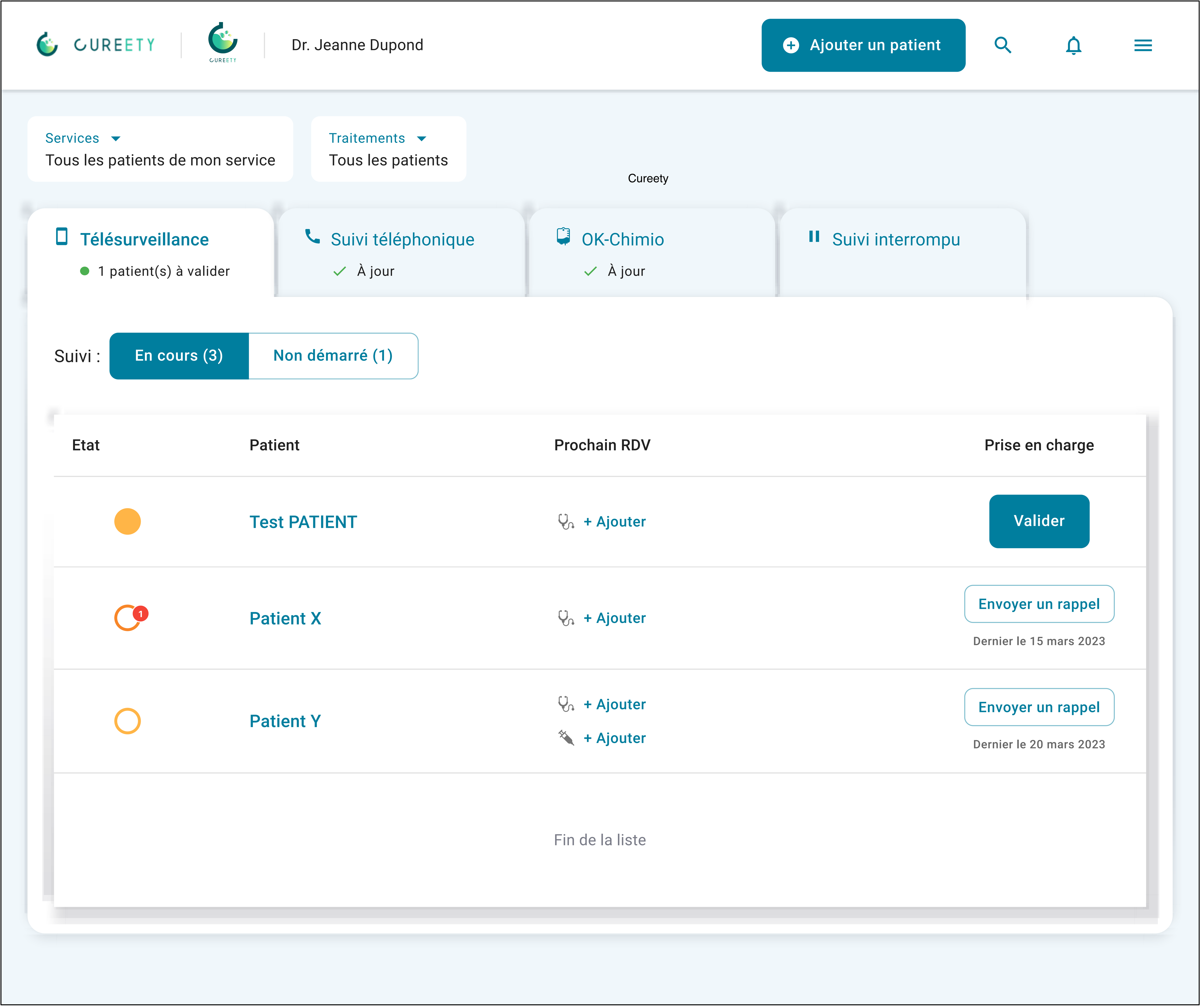

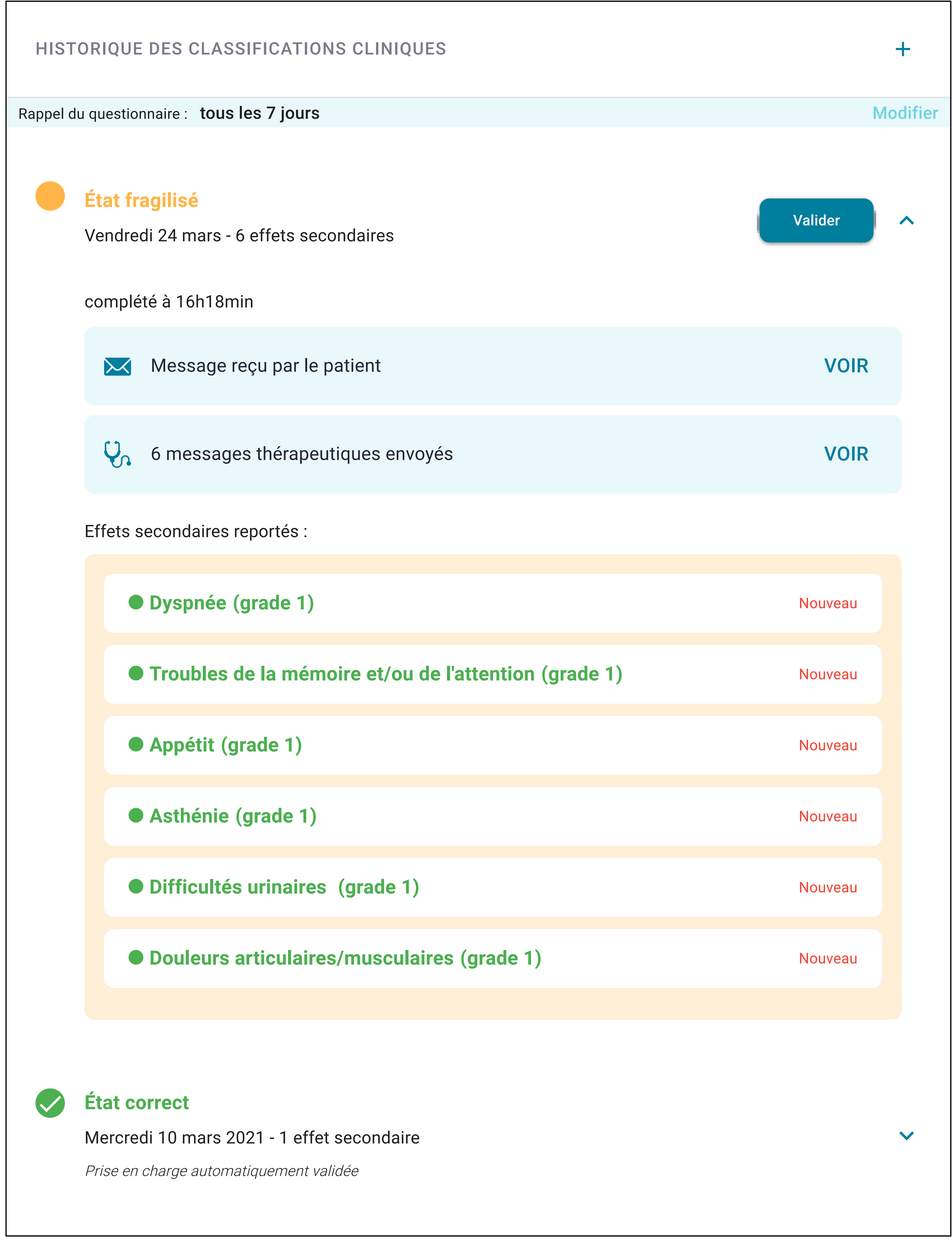

Supplementary Figure 2.
User interface of the Cureety platform for the medical team, showing an example of the patient dashboard with the latest clinical classifications for all the monitored patients, and then a detail of the classification and the AEs for a specific patient in a separate screen.
